# Daily Level Association of Physical Activity and Performance on Ecological Momentary Cognitive Tests in Free-living Environments: A Mobile Health Observational Study

**DOI:** 10.2196/33747

**Published:** 2022-01-31

**Authors:** Zvinka Z Zlatar, Laura M Campbell, Bin Tang, Spenser Gabin, Anne Heaton, Michael Higgins, Joel Swendsen, David J Moore, Raeanne C Moore

**Affiliations:** 1 Department of Psychiatry University of California, San Diego La Jolla, CA United States; 2 San Diego Joint Doctoral Program in Clinical Psychology San Diego State University/University of California San Diego, CA United States; 3 Department of Counseling and Marital and Family Therapy University of San Diego San Diego, CA United States; 4 Herbert Wertheim School of Public Health and Human Longevity Science University of California San Diego La Jolla, CA United States; 5 National Center for Scientific Research University of Bordeaux Bordeaux France; 6 Ecole Pratique des Hautes Etudes PSL Research University Paris France

**Keywords:** smartphones, neuropsychology, ecological momentary assessment, digital health, exercise, people living with HIV, aging, wearables, mobile cognition, mobile phone

## Abstract

**Background:**

Research suggests that physical activity (PA) has both acute and chronic beneficial effects on cognitive function in laboratory settings and under supervised conditions. Mobile health technologies make it possible to reliably measure PA and cognition in free-living environments, thus increasing generalizability and reach. Research is needed to determine whether the benefits of PA on cognitive function extend from the laboratory to real-world contexts.

**Objective:**

This observational study aims to examine the association between daily fluctuations in PA and cognitive performance using mobile health technologies in free-living environments.

**Methods:**

A total of 90 adults (mean age 59, SD 6.3 years; 65/90, 72% men) with various comorbidities (eg, cardiovascular risk and HIV) and different levels of baseline cognition (ranging from cognitively normal to impaired) completed ecological momentary cognitive tests (EMCTs) on a smartphone twice daily while wearing an accelerometer to capture PA levels for 14 days. Linear mixed-effects models examined the daily associations of PA with executive function and verbal learning EMCTs. Moderation analyses investigated whether the relationship between daily PA and daily performance on EMCTs changed as a function of baseline cognition, cardiovascular risk, and functional status (independent vs dependent).

**Results:**

Days with greater PA were associated with better (faster) performance on an executive function EMCT after covariate adjustment (estimate −0.013; *β*=−.16; *P*=.04). Moderation analyses (estimate 0.048; *β*=.58; *P*=.001) indicated that days with greater PA were associated with better (faster) executive function performance in individuals who were functionally dependent (effect size −0.53; *P*<.001) and not in functionally independent adults (effect size −0.01; *P*=.91).

**Conclusions:**

EMCTs may be a sensitive tool for capturing daily-level PA-related fluctuations in cognitive performance in real-world contexts and could be a promising candidate for tracking cognitive performance in digital health interventions aimed at increasing PA. Further research is needed to determine individual characteristics that may moderate the association between daily PA and EMCT performance in free-living environments.

## Introduction

### Background

The exponential growth of the older adult population will result in more individuals living with Alzheimer’s disease and related dementias (ADRD) [1]. Given the lack of effective treatments for Alzheimer’s disease, a focus on healthy lifestyle choices holds promise in preventing cognitive decline [2-4]. Evidence links physical activity (PA) with long-term cognitive benefits such as reduction of age-related cognitive decline and dementia risk. For example, a meta-analysis of longitudinal observational studies with middle-aged and older adults found that higher levels of PA were associated with a 14% lower risk of dementia when compared to those with lower levels of PA [5]. Similarly, compared with sedentary individuals, even low to moderate PA has been associated with a 35% reduction in the risk of cognitive decline [6]. Moreover, a recent review [7] found a small positive effect of PA on executive function (Cohen’s *d*=0.27) and memory (Cohen’s *d*=0.24), whereas another study found that walking more steps per day was related to better executive function in healthy aging [8]. Several mechanisms explain how PA benefits cognition in the long term, including angiogenesis, neurogenesis, upregulation of neurotrophic factors, reduced inflammation, cardiovascular benefits, changes in central arterial stiffness and endothelial function, and insulin regulation [9,10].

Less is known about the acute effects of PA on cognition in middle-aged and older adults in real-world contexts, where ecological validity is optimized. The acute effects of PA on cognition have generally been studied in laboratory settings, where individuals complete cognitive tasks before, during, or immediately following an exercise challenge. Within these settings, it has been found that acute moderate intensity PA results in improved executive function [11-13] and working memory performance in older adults [14], although others have found that acute exercise before memory encoding tasks may impair performance in older adults [15]. A meta-analysis of the literature showed that the overall effect of acute exercise on cognition is positive but generally small; and that longer exercise duration, greater intensity, type of cognitive performance assessed (and when it is assessed), and greater fitness level appear to be significant moderators of larger effect sizes [16].

Owing to research-grade accelerometry, it is now possible to remotely track PA behavior, whereas smartphone-based technology can assess cognitive function in real-world contexts using ecological momentary cognitive tests (EMCTs). Evidence suggests that EMCTs have relatively high completion rates of 60% to 85% [17], and performance on these tests correlates with standard neuropsychological testing scores [18-22]. With 81% of adults aged 60 to 69 years and 62% of those aged ≥70 years owning a smartphone [23,24], EMCTs can be deployed to increase our understanding of whether acute fluctuations in real-world PA lead to immediate improvements in cognition in a variety of everyday contexts. This can help determine the utility of EMCTs as tools in digital health interventions to measure cognitive function repeatedly, at scale, and in people’s natural environments, thus improving ecological validity and reducing participant burden.

### Objective

This observational study investigates if there are daily-level associations between accelerometer-measured PA and smartphone-based EMCT performance during a 2-week measurement period in middle-aged and older adults with a variety of comorbid conditions (eg, people living with HIV [PWH], cardiovascular risk, and functional impairment). Moreover, we examine whether cognitive status, functional status, and cardiovascular disease (CVD) risk affect the acute associations of PA with performance on mobile verbal learning and executive function EMCTs. We hypothesize that days with greater measured PA would be associated to better performance on executive function and learning. Consistent with the literature suggesting that those with greater risk profiles may benefit most from lifestyle approaches to maintain brain health [25], we also hypothesize that the association between daily fluctuations in PA and EMCT performance would be stronger in participants with lower cognition, higher CVD risk, and lower functional independence status compared to those with better cognition, lower CVD risk, and no functional dependence.

## Methods

### Participants

A total of 90 people—57 (63%) PWH and 33 (37%) HIV-negative, middle-aged, and older adults aged 50 to 74 years—were enrolled in a study at the University of California San Diego’s HIV Neurobehavioral Research Program (HNRP) from 2016 to 2019.

### Recruitment

Participants were recruited from the participant pool at the HNRP or through community outreach. Inclusion criteria were age ≥50 years, fluent in English, and ability to provide written informed consent. Exclusion criteria were neurological confounders not related to HIV (eg, stroke, untreated seizure disorder, and head injury with loss of consciousness >30 minutes), diagnosis of serious mental illness (eg, schizophrenia and bipolar disorder), and a reported learning disability or low estimated verbal IQ (ie, a standard score <70 on the Wide Range Achievement Test 4 Reading test [26]). The overall level of illicit substance use in this sample was low [27]. Illicit substance use was not exclusionary; however, participants with a positive alcohol breathalyzer or positive urine toxicology (other than marijuana or prescription medications) on the day of testing were rescheduled for another day. This occurred 2 times, and these 2 participants were rescheduled and tested positive once more upon their return, at which time they were withdrawn from the study. The study procedures were approved by the institutional review board of the University of California, San Diego, and all participants demonstrated decisional capacity [28] and provided written informed consent.

### Measures and Procedures

#### Study Overview

The study comprised a baseline in-person visit, a 14-day period of smartphone-based EMCTs and wrist-worn accelerometer tracking in participants’ natural environments, and a follow-up in-person visit. Participants were not co-enrolled in other research during the study period. Participants were compensated for the in-person assessments, for each EMCT that they completed, and for returning the study-owned smartphones and accelerometer watches. During the baseline visit, participants were given a wrist-worn accelerometer to track PA and a smartphone (Samsung Galaxy S 4.2 YP-GII) on which daily cognitive tests were administered. They received a 20- to 30-minute tutorial with an examiner on the use of the smartphone and how to complete the EMCTs. To ensure security of the data, the study phone’s operating system was encrypted in case the smartphone was lost or stolen. A user manual was sent home with participants, which included information on using the smartphone, proper wear of the accelerometer, frequently asked questions, and study staff contact information.

#### Baseline Laboratory Visit

##### Evaluation of Baseline Cognition

At the baseline visit, all participants completed a standardized and comprehensive neuropsychological battery used in research studies at the HNRP [29] covering 7 cognitive domains, including verbal fluency, speed of information processing, learning, delayed recall, working memory, executive function, and motor skills (Multimedia Appendix 1). Raw scores on these cognitive assessments were converted to practice effect–corrected scaled scores (mean 10, SD 3) to control for prior exposure to neuropsychological testing [30]. Next, scaled scores were converted to demographically adjusted (age, sex, education, and race) T scores for each test (T score <40 indicates impaired performance). Adjusted T scores were averaged to compute the global T score [31,32], which was the variable of interest in later analyses.

##### Evaluation of Functional Status

Participants completed the *Lawton–Brody instrumental activities of daily living* (IADL) questionnaire [33] to assess their ability to function independently in everyday life. The measure requires the participants to rate their ability to complete various basic (eg, bathing and dressing) and instrumental (eg, managing finances and cooking) activities of daily living both at their current level and *best ever* level of functioning. A decline in functioning is indicated if the current level of functioning is lower than that of the *best level* on any task. A participant with ≥2 declines is deemed *IADL-dependent*.

##### Evaluation of Cardiovascular Risk

To measure cardiovascular risk, we calculated the participants’ *Framingham CVD risk* scores [34]. This CVD risk score assigns weighted point values to the following factors: age, diabetes status, smoking status, systolic blood pressure (treated vs untreated), total cholesterol, and high-density lipoprotein cholesterol level to create a risk estimate score. The equation for calculating the Framingham CVD risk score is provided in the study by D’agostino et al [34].

##### Neuromedical Evaluation

During the baseline laboratory visit, participants completed a neuromedical evaluation, which included a fasting blood draw, in which an HIV and hepatitis C virus antibody point-of-care rapid test (Miriad-MedMira) was conducted and confirmed with western blot analyses. Blood samples were used to measure the current CD4 T-cell counts and plasma HIV viral loads (detectable at >50 copies/mL). All participants completed a neuromedical interview, including the collection of medical history, medication lists, and other HIV disease characteristics (ie, AIDS status, estimated duration of HIV disease, nadir CD4 T-cell count, and history of antiretroviral therapy).

#### At-home Monitoring: 14-Day Mobile Assessment

##### PA Measurement

To measure objective PA, participants wore the ActiGraph GT9X Link device (ActiGraph Inc) continuously on their nondominant wrist, except while bathing or swimming, for the duration of the 14-day EMCT period. Participants were also asked to record when and why they took the device off. The ActiGraph Link (ActiGraph Inc) is a triaxial accelerometer that has consistently been shown to be a valid and reliable measure of PA [35-37]. The wear location and assessment period are aligned with the best practices for PA assessment, resulting in high levels of acceptability and compliance among participants [38-41]. The device is small and lightweight and has a minimum of 512 MB of nonvolatile flash memory. The data are stored on the device. When participants returned the device, the ActiGraph data were immediately downloaded and screened by hour for completeness and possible irregularities or malfunction according to best practice recommendations [37,39,40]. Participants’ data were included if they wore the device for a minimum of 5 days, with at least 600 minutes of wear each day. Participants who achieved 4 days of wear, with at least 3000 total minutes, were also included. PA was defined as cumulative activity *counts* (ActiGraph’s proprietary metric) per day. This metric incorporates intensity, frequency, and duration of acceleration and is recommended for assessing the total volume of PA in a 24-hour period [42]. Specifically, counts are a result of summing the postfiltered accelerometer values (raw data at 30 Hz) into epoch *chunks*. The value of the counts varies based on the frequency and intensity of the raw acceleration. Vector magnitude was then calculated using the following equation:



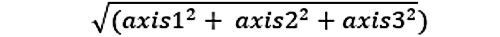



*Counts per minute* (CPM) is the average amount of total movement throughout the day; the higher the CPM, the more activity throughout the day. CPM is used as there are no established cutoff points for measuring sedentary, light, or moderate-to-vigorous PA at the wrist. Assessing the total volume of PA via vector magnitude CPM is important as it takes the frequency, intensity, and duration of activity bouts and condenses them down to a single metric that can be harmonized across studies.

##### EMCT Paradigm

**Figure 1:**
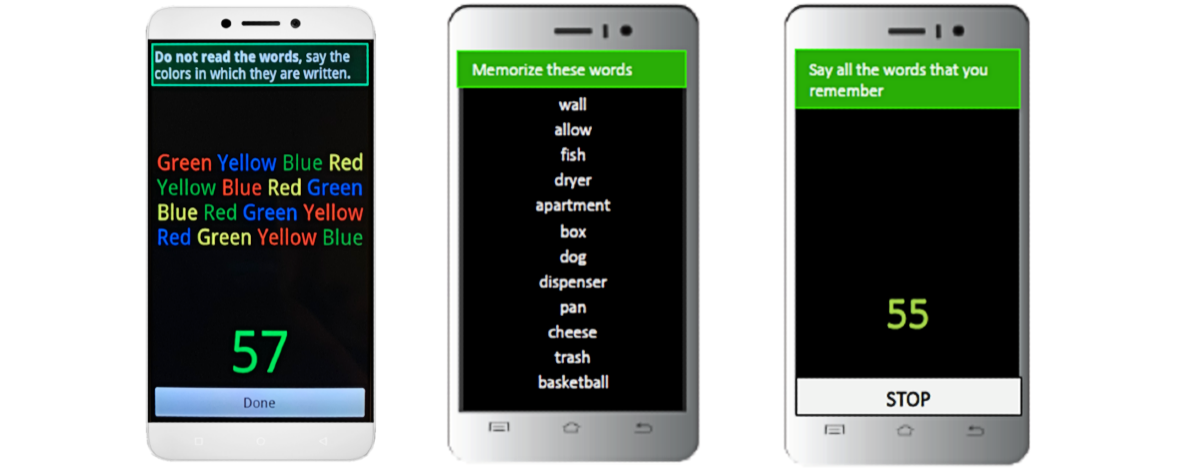
Example screenshot of the mobile color–word interference test (mCWIT; test of executive function; left panel) and the mobile verbal learning test (mVLT; test of verbal learning; right panel). The words on the mVLT are sample words and not an actual word list from the mVLT. All 12 words are presented on the screen, eliminating a need to scroll to view all the words.

An alarm sounded on the study smartphone twice a day for the 14-day assessment period to signal when it was time to complete 1 of the 2 mobile cognitive tests: once for the mobile color–word interference test (mCWIT) [18] and once for the mobile verbal learning test (mVLT; Figure 1) [19]. The alarms occurred at pseudorandom times throughout the day, accounting for participants’ preferred sleep-wake schedules, such that the participant did not know when they would be asked to complete the tests. Alarms occurred 2 to 3 hours apart. Once the alarm sounded, participants had 10 minutes to start the assessment (with a reminder alarm every 2 minutes during that 10-minute window) before it would time-out and be considered missed. Participants also had the option to cancel the survey during the 10-minute window or at any point during the survey. The mCWIT and mVLT were never given at the same time point.

The mCWIT is a test based on the Stroop paradigm assessing executive function. A total of 16 words (4 rows of 4 words) are presented in a different color than the printed word, and participants are instructed, “Do not read the words, say the colors in which they are written.” There was one trial for this test at each administration, for which participants had up to 60 seconds to say the colors for all 16 items as fast as possible. Each administration of the mCWIT alternated the order of words as well as the nonmatching color of each word. Responses were audio recorded and scored by 2 independent raters. All discrepant scores were assessed by a third rater. The outcome assessed in this study was the completion time (seconds).

The mVLT is a test designed to assess verbal learning and recall. Participants were presented with a list of 12 semantically unrelated words to read on the smartphone for three 30-second learning trials during which participants were told to memorize the words. After each of the 3 learning trials, participants were asked to immediately recall as many words as possible by saying them out loud into the phone. Participants had 60 seconds to recall words before the next trial began. A unique list was presented each day. Responses were audio recorded and scored for the total number of correct responses. The outcome assessed in this study was the total number of words correctly (out of a possible 36 words) recalled over the 3 learning trials per day. The mVLT was scored by 2 independent raters, and discrepant scores were reviewed by an additional rater.

For both the mCWIT and the mVLT, trials were excluded from analyses if raters suspected cheating (eg, help from others) or if the participant was doing something else during the test (eg, talking with others). mCWIT data were excluded from analyses for 2% (2/90) of participants; one of the participants was excluded because of colorblindness, and another participant was excluded as they had an average of 15/16 errors, and therefore, their data were not considered valid. There were also 2% (2/90) of participants who had 14% mCWIT compliance (the minimal compliance threshold of 30% was set for analyses) and were thus removed from the analyses.

### Statistical Analysis

Demographic data, comorbid conditions, and HIV disease characteristics were summarized as mean (SD), median (IQR), or n (%). mCWIT in seconds was log_10_-transformed to improve normality before the analyses. To evaluate whether days with greater PA were associated with better EMCT performance, linear mixed-effects models with subject-specific random intercepts were conducted to detect the relationship between within-person PA and EMCT performance. Between-person PA effects on EMCT performance were also estimated. Crude models with significant within- or between-person effects (*P<*.05) were later adjusted for covariates. HIV status, age, sex, education, and race or ethnicity (non-Hispanic White vs all other race or ethnicities) were included as covariates in the models if *P*<.10, using backward model selection.

In addition, to determine whether cognition (ie, global T score), functional status (ie, IADL dependent or independent), and CVD risk (Framingham CVD risk profile) moderated the relationship between PA and EMCT performance, moderation analyses were conducted using separate linear mixed-effects models with fixed effects of within- and between-person PA, global T score, and IADL status or Framingham stroke risk score; their interactions (eg, an interaction between within-person PA and global T score on EMCT performance); and subject-specific random effects. All models were controlled for study day. The significance level of α was set at .05. The effect sizes (and 95% CIs) are standardized coefficients, which are analogous to Cohen’s *d*. R software (version 3.6.0; R Foundation for Statistical Computing) was used to perform all statistical analyses.

## Results

### Participants

Participants’ demographic, clinical, and cognitive characteristics are presented in Table 1. On average, participants were in their late 50s (mean age 59, SD 6.3 years), had some college education (mean 14.4, SD 2.4 years), and most were male (65/90, 72%) and identified as non-Hispanic White (57/90, 63%). Of the 90 participants, 57 (63%) were PWH, and participants’ age, education, and race or ethnicity were not significantly different based on HIV status. There were significantly more women in the HIV-negative group compared with the PWH group (PWH: 10/57, 18% women; HIV-negative: 15/33, 45% women; *P*<.004). Overall, there was excellent adherence to the mobile cognitive testing and accelerometer protocols as, on average, participants completed 13 of 14 days of the mCWIT, 12 of 14 days of the mVLT, and had 12 of 14 days of accelerometer data to objectively assess PA.

**Table 1:** Participant characteristics (N=90).

Demographic variables		Values
Age (years), mean (SD)		59.0 (6.3)
Male, n (%)		65 (72)
**Race and ethnicity, n (%)**		
	Non-Hispanic White	57 (63)
	African American or Black	19 (21)
	Hispanic or Latino	11 (12)
	Other	3 (3)
Education (years), mean (SD)		14.4 (2.4)
**Comorbid conditions**		
	Hyperlipidemia, n (%)	52 (58)
	Hypertension, n (%)	50 (56)
	Diabetes mellitus, n (%)	21 (23)
	BMI (n=80), median (IQR)	27.0 (25.1-32.5)
	Framingham cardiovascular disease risk score (n=80), median (IQR)	16.1 (9.4-27.33)
	Depression (Beck Depression Inventory-2), median (IQR)	4 (1-10)
	Lifetime any substance use disorder, n (%)	57 (63)
	Current substance use disorder^a^, n (%)	3 (3)
**HIV characteristics^b^**		
	AIDS, n (%)	39 (68)
	Current CD4, median (IQR)	690 (549-879)
	Nadir CD4, median (IQR)	145 (35-300)
	Duration of HIV infection (years), median (IQR)	25.2 (18.5-28.9)
	On antiretroviral therapy, n (%)	53 (93)
	Undetectable viral load (n=53), n (%)	52 (98)
**Cognitive variables**		
	Global T score, mean (SD)	49.5 (6.2)
	Independent activities of daily living: dependent, n (%)	30 (33)
	Mobile color–word interference test score (seconds; n=88), mean (SD)	23.4 (6.1)
	Number of mobile color–word interference test trials completed (n=88), median (IQR)	13 (11-13.25)
	Mobile verbal learning test score (total correct), mean (SD)	18.9 (4.8)
	Number of mobile verbal learning test trials completed, median (IQR)	12 (11-13.25)
**Physical activity**		
	Average vector magnitude counts per minute/day, mean (SD)	2047 (616.7)
	Wear time (days), median (IQR)	12 (11-14)

^a^All current substance use disorders were cannabis or alcohol use disorders.

^b^Approximately 63% (57/90) were HIV-positive.

### Within- and Between-Person Associations of PA and EMCT Performance

#### Overview

Results and effect sizes are presented in Table 2.

**Table 2:** Mixed-effects models for associations between physical activity and ecological momentary cognitive testing performance.

Models			Estimate (95% CI)	Effect size^a^ (95% CI)	*P* value
**Mobile color–word interference test of executive function (log_10_ transformed)**					
	**Model 1 crude**				
		Within-person physical activity^b^	−0.013 (−0.0025 to −0.00005)	−0.15 (−0.30 to 0.0004)	.049
		Between-person physical activity	−0.013 (−0.048 to 0.023)	−0.15 (−0.59 to 0.29)	.50
	**Model 1 adjusted^c^**				
		Within-person physical activity	−0.013 (−0.026 to −0.0008)	−0.16 (−0.31 to −0.008)	.04
		Between-person physical activity	−0.004 (−0.038 to 0.030)	−0.049 (−0.48 to 0.38)	.82
**Mobile verbal learning test**					
	**Model 2 crude^d^**				
		Within-person physical activity	−0.52 (−1.37 to 0.33)	−0.093 (−0.25 to 0.061)	.24
		Between-person physical activity	0.051 (−1.53 to 1.64)	0.009 (−0.28 to 0.29)	.95

^a^Standardized regression coefficient analogous to Cohen’s *d*.

^b^Within-person and between-person physical activity is reflected as counts per minute/1000. All analyses control for study day.

^c^Adjusted for HIV status, age (in years), and race or ethnicity (reference: non-Hispanic White); the interaction between HIV status and physical activity was not significant and was therefore removed from the adjusted model.

^d^Model 2 was not subsequently adjusted for covariates because of the crude model being nonsignificant.

#### mCWIT Test of Executive Function Model 1 Crude

Within-person PA was significantly associated with mCWIT performance such that greater daily PA was associated with faster (ie, better) daily mCWIT performance. The between-person association of PA and mCWIT was not significant, indicating that average PA was not significantly associated with average mCWIT performance in this sample.

#### mCWIT Test of Executive Function Model 1 Adjusted

**Figure 2:**
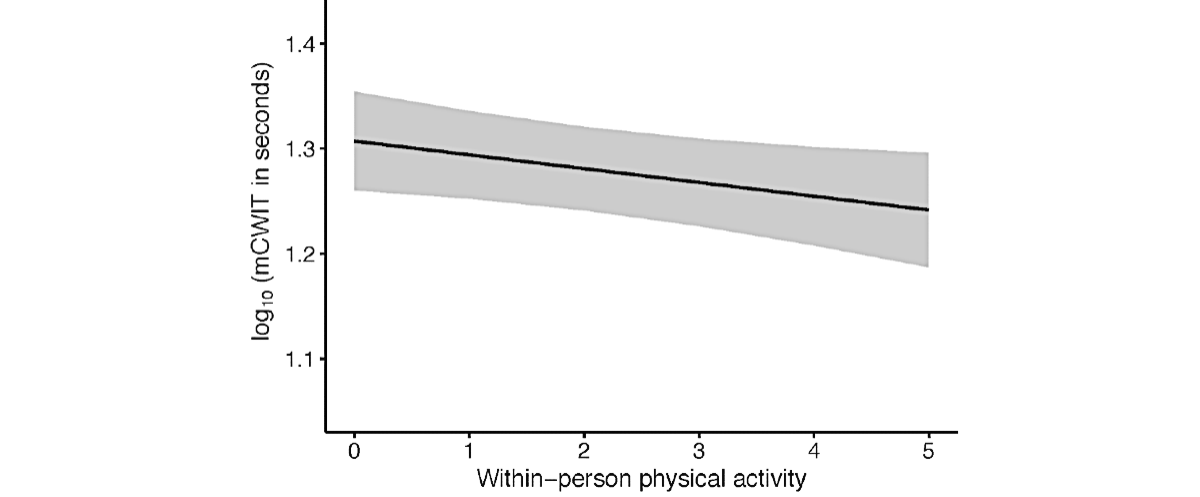
Greater daily physical activity was associated with faster daily mobile color-word interference test of executive function performance. Within-person physical activity is reflected as counts per minute/1000. Variables were adjusted for between-person physical activity, HIV status, age, ethnicity, and study day. Shaded bands represent 95% CIs. mCWIT: mobile color-word interference test.

The significant within-person effect held after accounting for HIV status and the demographic variables that significantly improved the model (ie, age and race or ethnicity). The results indicate that for every increase of 1 SD in PA, mCWIT performance (on the log_10_ scale) improved (was faster) by 0.16 SDs, or mCWIT performance in seconds decreased (was faster) by 3% for every 1000-unit increase in PA (Figure 2). As with the crude model, the between-person association of PA and mCWIT was not significant.

To determine whether the significant within-person effects varied by HIV status, we examined the interaction term of HIV status by within-person PA. The interaction term did not reach significance (estimate 0.002; *P*=.88; effect size −0.025), indicating that the within-person association between daily PA and daily mCWIT performance did not differ by HIV status. Therefore, the HIV by PA interaction was not included in subsequent models examining the mCWIT.

#### mVLT Test of Verbal Learning Model 2 Crude

Neither the within nor the between-person effects of PA with mVLT performance were significant, indicating that neither daily nor average PA was related to mVLT performance.

### Sensitivity Analyses: Global Cognition, Functional Status, and Cardiovascular Risk

#### Overview

The results of the sensitivity analyses are presented in Table 3. We examined whether global cognition (ie, global T score), functional status (ie, IADL status), and CVD risk (ie, Framingham CVD risk score) moderated the relationship of within- or between-person PA and mobile cognitive test performance. Only models with significant interaction terms were adjusted for covariates.

**Table 3:** Mixed-effects models to examine whether cognition (global T score), Lawton–Brody instrumental activities of daily living (IADL), and cardiovascular risk (Framingham cardiovascular disease [CVD] risk score) moderated the association of physical activity (PA) and ecological momentary cognitive testing performance.

Model			Estimate (95% CI)	Effect size^a^ (95% CI)	*P* value
**mCWIT^b^ test of executive function (log_10_ transformed)**					
	**Model 3: global T score**				
		Global T score	−0.002 (−0.015 to 0.010)	−0.030 (−0.18 to 0.12)	.70
		Within-person PA^c^×global T score	−.0007 (−0.003 to 0.001)	−0.008 (−0.033 to 0.017)	.52
		Between-person PA×global T score	−.003 (−0.008 to 0.003)	−0.031 (−0.100 to 0.039)	.39
	**Model 4 crude: IADL^d^**				
		IADL status (reference: dependent)	0.046 (−0.12 to 0.21)	0.57 (−1.45 to 2.59)	.58
		Within-person PA×IADL status	0.049 (0.021 to 0.077)	0.60 (0.26 to 0.95)	.001
		Between-person PA×IADL status	−0.026 (−0.10 to 0.049)	−0.32 (−1.25 to 0.62)	.51
	**Model 4 adjusted^e^: IADL**				
		IADL status (reference: dependent)	0.018 (−0.14 to 0.17)	0.22 (−1.68 to 2.12)	.82
		Within-person PA IADL status	0.048 (0.019 to 0.075)	0.58 (0.24 to 0.92)	.001
		Between-person PA×IADL status	−0.005 (−0.074 to 0.064)	−0.065 (−0.94 to 0.81)	.89
	**Model 5: Framingham CVD risk score**				
		CVD risk	0.003 (−0.004 to 0.011)	0.038 (−0.054 to 0.13)	.42
		Within-person PA×CVD risk	0.0002 (−0.0008 to 0.001)	0.002 (−0.009 to 0.014)	.70
		Between-person PA×CVD risk	−0.001 (−0.005 to 0.002)	−0.016 (−0.062 to 0.030)	.50
**mVLT^f^ test of verbal learning**					
	**Model 6: global T score**				
		Global T score	0.49 (−0.078 to 1.06)	0.088 (−0.016 to 0.19)	.10
		Within-person PA×global T score	−0.042 (−0.18 to 0.090)	−0.008 (−0.032 to 0.016)	.54
		Between-person PA×global T score	−0.13 (−0.39 to 0.13)	−0.023 (−0.070 to 0.025)	.35
	**Model 7: IADL**				
		IADL status (reference: dependent)	5.59 (−1.49 to 12.7)	1.00 (−0.29 to 2.29)	.13
		Within-person PA×IADL status	0.67 (−1.30 to 2.63)	0.12 (−0.23 to 0.47)	.50
		Between-person PA×IADL status	−2.60 (−5.90 to 0.69)	−0.47 (−1.07 to 0.13)	.13
	**Model 8: Framingham CVD risk score**				
		CVD risk	0.068 (−0.26 to 0.40)	0.012 (−0.048 to 0.073)	.69
		Within-person PA×CVD risk	0.009 (−0.058 to 0.079)	0.002 (−0.011 to 0.014)	.79
		Between-person PA×CVD risk	−0.052 (−0.22 to 0.11)	−0.010 (−0.040 to 0.021)	.54

^a^Analogous to Cohen’s *d* (standardized regression coefficient).

^b^mCWIT: mobile color–word interference test.

^c^Within-person and between-person PA was reflected as counts per minute/1000. All analyses control for study day.

^d^Lawton–Brody instrumental activities of daily living questionnaire.

^e^Adjusted for HIV status, age (in years), and race or ethnicity (reference: non-Hispanic White).

^f^mVLT: mobile verbal learning test.

#### Relationships With Cognitive Global T Score

When predicting the mCWIT, the within-person PA by cognitive global T score and the between-person PA by cognitive global T score interactions were not significant (Table 3). When removing interaction terms, within-person PA remained significantly associated with mCWIT performance after accounting for cognitive global T score (estimate −0.013; *P*=.04). Examining the mVLT, neither the within-person PA by cognitive global T score nor the between-person PA by cognitive global T score interactions were significant.

#### Relationships With IADL Status

**Figure 3:**
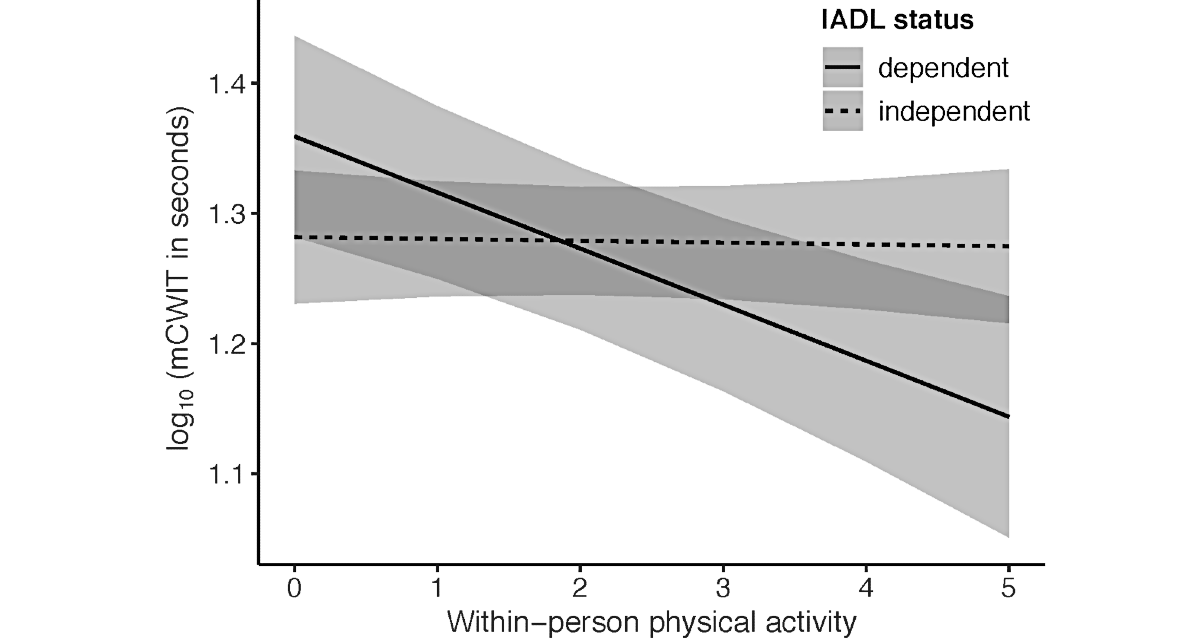
Instrumental activities of daily living moderate the relationship of within-person physical activity and mobile color–word interference test of executive function performance. Within-person physical activity is reflected as counts per minute/1000. Variables were adjusted for between-person physical activity, HIV status, age, ethnicity, and study day. Shaded bands represent 95% CIs. IADL: instrumental activities of daily living; mCWIT: mobile color-word interference test.

There was a significant within-person PA by IADL score interaction effect on mCWIT performance. The relationship between greater daily PA and better mCWIT performance was significant for those who reported IADL dependence (effect size −0.53; *P*<.001) and not for those who were independent in IADL (effect size −0.01; *P*=.91; Figure 3). For those who reported IADL dependence, mCWIT performance decreased on average by 9.6% for every 1000 units of within-person PA. The interaction term remained significant when adjusting for demographic variables that significantly improved model fit (ie, age and race) and HIV status. The between-person PA by IADL score interaction was not significantly associated with mCWIT performance. Neither the between-person nor the within-person PA by IADL score interactions were significantly associated with mVLT performance.

#### Relationships With Framingham CVD Risk Score

For the mCWIT, the cardiovascular risk by within- and between-person PA interactions were not significant. After removing the interactions, within-person PA remained significantly related to mCWIT performance, even when accounting for cardiovascular risk (estimate −0.014; *P*=.048). When examining the mVLT, neither the within- nor between-person PA by cardiovascular risk interactions was significant.

## Discussion

### Principal Findings

We examined the cross-sectional association of objectively measured daily PA with EMCT performance to determine whether natural variation in PA was accompanied by fluctuations in performance on EMCTs of executive function and verbal learning in individuals’ free-living environments. In a sample of diverse adults with a wide range of comorbidities (ie, cardiovascular risk and HIV), we found that during days with greater PA (ie, a combination of higher frequency, intensity, and duration), participants had faster (better) performance on a mobile Stroop-like task of executive function (mCWIT). This relationship persisted after adjusting for HIV status and other demographics (age and race or ethnicity). No effects of PA were found on a task of verbal learning (mVLT). The results are consistent with much of the laboratory-based literature linking an acute increase in PA to improvements in executive functions [11-13] and extending it to provide novel evidence that these associations may be captured remotely in the real world using EMCTs and accelerometry.

Next, we examined whether the significant cross-sectional association of daily PA with EMCT performance varied as a function of cognitive status, functional status (IADL), and cardiovascular risk, all of which elevate the risk for ADRD [43-47]. We found that only for individuals who reported being functionally dependent on IADL, there was a significant association between daily PA and executive function performance. It has been suggested that individuals who are at higher risk of ADRD may benefit most from lifestyle interventions to reduce cognitive decline [25]. Our findings are in line with this literature, suggesting that those with more functional limitations may see a greater cognitive benefit from engagement in PA in their free-living environments. We did not find moderation effects related to HIV, cognitive, or cardiovascular risk status, suggesting that the daily relationship of PA with executive function does not significantly vary as a result of these risk factors in this limited sample of adults. Importantly, the association of daily PA with daily executive function performance persisted after adjusting these models for HIV status, global cognition, and cardiovascular risk, reinforcing the robustness of this relationship in a heterogeneous sample.

The findings suggest that daily variation in PA is sensitive to fluctuations in daily executive function performance, which can be reliably measured remotely using actigraphy and EMCTs. This has important implications for the development of novel digital health interventions to lower the risk of ADRD. Given the many limitations preventing individuals from participating in supervised or group-based PAs (ie, transportation barriers, safety issues, and mobility limitations) and the high costs associated with in-person interventions, it is important to develop scalable, low-cost, evidence-based [48] interventions to promote PA in real-world contexts [49]. Digital health technologies can help us achieve this goal by tracking intervention adherence and providing feedback in real time [50]. Future lifestyle interventions to preserve brain health can leverage these technologies to measure PA and cognition in real-world contexts to better understand treatment effects, improve generalizability, and reach out to a larger sector of the population who may not otherwise be able to attend laboratory-based (in-person) clinical trial visits. Moreover, digital health technologies can help track intervention adherence and changes in outcomes of interest, such as cognition, which can be measured repeatedly over the course of a clinical trial rather than only at baseline and post intervention time points to better capture change over time.

### Limitations

A limitation of this study is its observational rather than interventional nature, which does not allow us to determine whether improvements in PA lead to better executive function performance or vice versa. Second, the measurement time was limited to 14 days, which may have limited the range of variability in PA and cognitive testing performance. Third, this study was performed on a small and heterogeneous sample of adults with various comorbid conditions, limiting our ability to adjust the models for other potential confounders that may have affected EMCT performance, such as mood and motivation indicators. That said, it is unlikely that our results would change if the models were adjusted for mood, given previous findings showing that adjusting for mood did not alter the significant association of daily activities with EMCT performance in this sample [51]. Moreover, given the small sample size, null findings may have resulted from a lack of power rather than an absence of associations. Fourth, accelerometers were worn on the wrist rather than the hip, which has been traditionally thought to provide a more accurate measurement of PA [52], although this assumption has recently been challenged, given the ubiquitous nature of wrist-worn accelerometry and their improved estimation of PA [40,53,54]. Fifth, participants were paid to complete the EMCTs; therefore, adherence may differ in studies where participants do not receive financial compensation for completing the assessments. Finally, future studies with larger sample sizes should account for more variables that may affect the relationship between PA and cognition, such as the length and severity of HIV disease.

### Conclusions

In conclusion, using EMCTs and accelerometry to capture cognitive performance and PA in free-living environments may be an ecologically valid means of capturing real-world associations between cognition and PA. Our findings suggest that these digital techniques are promising candidates for tracking cognitive change and may be useful in the context of lifestyle (nonpharmacological) digital interventions designed to reduce ADRD risk and improve brain and cognitive health. Having participants complete cognitive tests in more familiar settings, such as their home or during other daily activities, can help increase the generalizability of findings, reduce intervention costs, increase scalability, and improve adherence to digital health interventions.

## Appendix

Multimedia Appendix 1 HIV Neurobehavioral Research Program neuropsychology battery.

## Abbreviations

ADRD: Alzheimer’s disease and related dementias
CPM: counts per minute
CVD: cardiovascular disease
EMCT: ecological momentary cognitive test
HNRC: HIV Neurobehavioral Research Center
HNRP: HIV Neurobehavioral Research Program
IADL: instrumental activities of daily living
mCWIT: mobile color–word interference test
mVLT: mobile verbal learning test
NIMH: National Institute of Mental Health
PA: physical activity
PWH: people living with HIV
