# Supplementary material for: Daily Level Association of Physical Activity and Performance on Ecological Momentary Cognitive Tests in Free-living Environments: A Mobile Health Observational Study
Source: JMIR Mhealth Uhealth. 2022 Jan 31;10(1):e33747. doi: 10.2196/33747 (PMC8845015; doi:10.2196/33747)
Supplement: Multimedia Appendix 1 [file mhealth_v10i1e33747_app1.docx]

**Multimedia Appendix 1.** HIV Neurobehavioral Research Program neuropsychology battery.

| **Verbal Fluency** | **Learning** |
| --- | --- |
| Controlled Oral Word Association Test  (FAS) | Hopkins Verbal Learning Test-Revised (Total  Learning) |
| Category Fluency Test (“animals” and  “actions”) | Brief Visuospatial Memory Test-Revised  (Total Learning) |
| **Executive Function** | **Delayed Recall** |
| Wisconsin Card Sorting Test  (Computerized 64-cards) | Hopkins Verbal Learning Test-Revised  (Delayed Recall) |
| Trail Making Test Part B | Brief Visuospatial Memory Test-Revised (Delayed  Recall) |
| Stroop Color and Word Test  (Interference score) | **Working Memory** |
| **Speed of Information Processing** | WAIS-III Letter-Number Sequencing |
| WAIS-III Digit Symbol | Paced Auditory Serial Addition Task |
| WAIS-III Symbol Search | **Complex Motor Skills** |
| Trail Making Test Part A | Grooved Pegboard Test (Dominant and Non-  Dominant) |
| Stroop Color and Word Test (color trial) |  |
